# MR guided focused ultrasound

**DOI:** 10.1186/1470-7330-15-S1-O31

**Published:** 2015-10-02

**Authors:** Wladyslaw Gedroyc

**Affiliations:** 1 Radiology Department Imperial College Healthcare NHS Trust, London, UK

## 

Focused ultrasound utilises powers of approximately 5000 to 10,000 times that of conventional diagnostic ultrasound which can be focused to a very small point in the tissues of the body where it produces rapid very intense heating which causes almost instantaneous ablation of heated tissues at the target site. No intervention is required and the whole process in this format is carried out within an MR scanner. MR allows excellent targeting of the site to be ablated and can provide an in vivo thermal map of heat delivery as it is deposited in the target tissue so that an accurate and very safe procedure can be performed as long as there is a suitable acoustic window available to reach the target site through the anterior tissues.

This lecture will describe the areas where MR guided focused ultrasound has been utilised and will describe areas of evolving technology and the technological problems that are encountered in these applications. Whilst some of the applications described such as the utilisation of focused ultrasound in fibroids and in the brain are not directly applications that treat malignancy the principles of these treatments can be applied to other malignant disease processes and will in the future allow malignancy to be treated at these sites.

The largest experience in the world in this therapy is available in the pelvis in the treatment of uterine fibroids and this procedure will be described. The 2^nd^ FDA approved application of MR guided focused ultrasound is in the treatment of bone secondaries and the utilisation of focused ultrasound this area and its problems and potential will be described. MR guided focused ultrasound shows immense promise in the treatment of liver tumours but the technological problems in this application are huge and these will be described briefly and the methods by which we hope to overcome these problems will be provided. Focused ultrasound utilisation in the treatment of prostate cancer is one of the oldest applications of this technology and new transducers are now available utilising MR guidance which will substantially improve the complications seen with all types of prostate cancer treatment by providing excellent targeting accuracy of the deposition of destructive energy within the prostate and avoiding the neurovascular bundles.

A brief description of the brain applications will be provided and although these are not malignant at the moment the future holds out significant promise in this field and this application is of great interest to all physicians in this field.

Drug activation with focused ultrasound will become a very large field of its own and a very brief description of how this may work and what technologies are required will be given although this field has not yet entered the full clinical arena and 1^st^ in human trials have not started yet but the promise that this type of approach provides is immense.

